# Citizens’ Actions in Response to Chikungunya Outbreaks, Réunion Island, 2006

**DOI:** 10.3201/eid2105.141385

**Published:** 2015-05

**Authors:** Bernard-Alex Gaüzère, Jean-Hugues Mausole, Fabrice Simon

**Affiliations:** Centre Hospitalier Universitaire, Saint-Denis, Reunion, France (B.-A. Gaüzère);; Association des Victimes de l’Épidémie de Chikungunya, Saint-Denis, France (J.-H. Mausole);; Laveran Teaching Military Hospital, Marseille, France (F. Simon)

**Keywords:** chikungunya, chikungunya virus, viruses, outbreaks, citizens’ actions, responses, Réunion Island

**To the Editor:** To get ready for the spread of chikungunya, health authorities in North, South, and Central America and the Pacific Islands are developing preparedness and response plans (*1*,*2*) that contain vector control, epidemiologic surveillance, medical education, and communication components. They might consider the experience of Réunion Island, an overseas department of France, where a chikungunya outbreak affected 38.5% of its 800,000 inhabitants during the first 3 months of 2006 (*3*). Although the island was unprepared to deal with such a massive outbreak (*4*), the disease was under control by the middle of 2006; only a few sporadic cases occurred during the following years. In addition to taking recommended public health measures, public health officials in France created a task force with physicians (including intensive care unit doctors, pediatricians, and obstetricians), specialists in public health and social sciences, virologists, immunologists, entomologists, and pathologists (*5*) to develop a multidisciplinary approach to the outbreak.

Some citizens’ initiatives complemented the official measures. First, associations of chikungunya virus–infected patients helped families (through means that included psychological and friendly support and home visits) and updated mass media with regard to disease complications, persistent symptoms, and administrative difficulties (including receiving long-term sick leave and disability, recognition of professional exposure, and free analgesic medication). Second, citizens created a chikungunya-dedicated website (http://www.chikungunya.net) that included citizens’ frequently asked questions and university-affiliated physicians’ responses and patients’ forums. Third, citizens actively supported the twice-yearly *Kass moustik* (Creole for “to break mosquitoes”) operations, which involved vast community mobilizations to educate persons on mosquitoes’ role in spreading chikungunya and to destroy breeding sites near homes. The operations also involved mobilizing community-based and municipality groups, making door-to-door visits, and lobbying for government funds (each operation cost US $60,000). After implementation of these initiatives, telephone operators sent health messages to all cell phones on the island. These actions demonstrate that citizens have a place in their countries’ response to chikungunya outbreaks.
